# Carded Cotton to Blistered Surfaces

**Published:** 1830-04-01

**Authors:** 


### CARDED COTTON TO BLISTERED SURFACES.

Dr. Merrill, of Nutchez, in America, communicates to the editor of the North American Medical and Surgical Journal, the good effects of cotton, when applied to blistered surfaces, immediately after the blister is removed. The layer of cotton should be half an inch, or more, in thickness, and sufficiently large to insure absorption of the discharge. In two days, under ordinary circumstances, a new cuticle will be formed, and the blister healed. This dressing gives no pain, and is very convenient in many cases, whether the patient be confined or not to the house.

---

